# Acute Onset Lactic Acidosis Secondary to Linezolid

**DOI:** 10.7759/cureus.35891

**Published:** 2023-03-08

**Authors:** Tara Kronen, Avni Agrawal, Pramod Reddy

**Affiliations:** 1 Internal Medicine, University of Florida College of Medicine, Jacksonville, USA

**Keywords:** hyper-lactatemia, zyvox, acidosis lactic, antibiotic side effects, linezolid induced lactic acidosis, adverse drug events, multi-drug resistant bacteria, anion-gap metabolic acidosis, linezolid

## Abstract

Linezolid has been the mainstay of treatment for multidrug-resistant Gram-positive bacteria. Common adverse effects with linezolid include diarrhea, nausea, headache, and bone marrow suppression. A less common and understudied side effect is lactic acidosis. This study describes a 19-year-old man with linezolid-induced lactic acidosis (LILA). The patient was admitted for the management of acute decompensated systolic heart failure, which improved on guideline-directed medication therapy (GDMT). During hospitalization, he developed an erythematous weeping cellulitis infection of his right lower extremity and was started on linezolid 600 mg every 12 hours with wound and blood cultures collected. After one day of treatment with linezolid, lactic acid levels acutely increased from 1.8 mmol/L to 5 mmol/L without any other interventions. Suspecting possible LILA, linezolid was transitioned to cephalexin with a reduction of lactic acid to 2.4 mmol/L, one day following linezolid cessation. After two days of linezolid cessation, lactic acid levels decreased to 1.9 mmol/L. Lactic acidosis can have profound hemodynamic consequences for patients, including death. A meta-analysis study of 35 articles with 47 patients (28 males, 18 females, and one non-binary) was done, which found a 25.5% mortality rate associated with LILA. Due to this high mortality, having a greater understanding of the associated risk factors with LILA is very important. This case study aims to inform clinicians of the potential harmful side effects associated with linezolid, as well as the understudied risk factors involved in LILA that are needed to prevent its occurrence.

## Introduction

Linezolid has been the mainstay of treatment for multidrug-resistant Gram-positive bacteria. Its mechanism of action inhibits the translation of bacterial proteins through the binding of bacterial 23S ribosomal ribonucleic acid of the 50S large subunit [1]. Some common adverse effects include diarrhea, nausea, headache, and bone marrow suppression. A less common and understudied side effect is lactic acidosis [2]. The proposed mechanism of linezolid-induced lactic acidosis (LILA) is believed to be through the prevention of mitochondrial-derived respiratory complexes, preventing adenosine triphosphate (ATP) formation [3]. Here, we present a case of short-term use of linezolid leading to lactic acidosis in order to inform clinicians of the potential harmful side effects associated with linezolid.

## Case presentation

This is a 19-year-old man, with a past medical history of dilated cardiomyopathy with an ejection fraction of 10-15% and morbid obesity, who was admitted for the management of acute decompensated systolic heart failure. On presentation, blood pressure was 143/85 mm Hg, heart rate was 104 beats/min, respiratory rate was 18 breaths/min, body temperature was 36.8°C, and oxygen saturation was above 95% on room air. Physical examination was notable for bilateral inspiratory rales, third heart sound (S3), jugular venous pressure, 3+ pitting peripheral edema, warm extremities, and 2+ pulses bilaterally. Laboratory data revealed elevated N-terminal prohormone of B-type natriuretic peptide (NT-proBNP) 3,715 pg/mL, creatinine (Cr) 0.82 mg/dL, and lactic acid 1.9 mmol/L. Chest radiography showed marked cardiomegaly with moderate pulmonary edema. The patient was started on bumetanide, lisinopril, and spironolactone. During hospitalization, the patient developed an erythematous weeping cellulitis infection of his right lower extremity. Lactic acid was collected along with wound and blood cultures. He was started on linezolid 600 mg every 12 hours. After one day of treatment with linezolid, lactic acid levels acutely increased from 1.8 mmol/L to 5 mmol/L without any other interventions. Due to acute increase in lactic acidosis without an identifiable cause, serial monitoring was initiated. On physical examination, the patient’s blood pressure dropped to 110/70 mm Hg with all other vital signs remaining stable. The patient was alert and oriented to name, place, time, and event. The lungs were clear to auscultation, and extremities were warm, well perfused with 2+ distal pulses, and showed improved 1+ pitting peripheral edema. Suspecting possible LILA, linezolid was transitioned to cephalexin. Lactic acid was subsequently reduced to 2.4 mmol/L. After two days of linezolid cessation, lactic acid levels decreased to 1.9 mmol/L (Table 1). No other medications were changed during this time period, and blood cultures remained negative. The patient was found to have a left ventricular thrombus, started on warfarin, and reached therapeutic international normalized ratio (INR) levels. Additionally, the patient had an implantable cardioverter defibrillator (ICD) placed due to low ejection fraction (EF). The patient was then stable for discharge.

**Table 1:** Summary of lactic acid, bicarbonate, anion gap, and creatinine values before, during, and after linezolid therapy. As illustrated on hospital days 1 and 3, the patient’s laboratory values were within normal limits prior to linezolid therapy. On day 4, lactic acid levels increased, bicarbonate levels decreased, and anion gap increased, with creatinine levels remaining close to baseline (shown in bold). This indicated that lactic acidosis was most likely caused by the induction of linezolid therapy. On hospital day 5, linezolid was stopped with laboratory values showing improvement (shown in bold). Lastly, on hospital day 6, after two days of linezolid cessation, laboratory values returned to baseline.

Hospital day	Day 1	Day 3	Day 4 (after one day of linezolid therapy)	Day 5 (after one day of linezolid cessation)	Day 6 (after two days of linezolid cessation
Lactic acid (mmol/L)	1.9	1.8	5	2.4	1.9
Bicarbonate (mmol/L)	26	24	20	24	24
Anion gap (mmol/L)	11	12	16	12	12
Creatinine (mg/dL)	0.82	0.81	0.84	0.85	0.82

## Discussion

Two types of lactic acidosis have been identified: Type A and Type B. Type A is due to hypoperfusion and hypoxia, leading to anaerobic glycolysis. Type B is not associated with tissue hypoxia or hypoperfusion and includes all other causes [4]. Our patient can be categorized with Type B lactic acidosis since other causes of lactic acidosis were excluded. The patient was hemodynamically stable, well perfused, and not in cardiogenic shock despite heart failure with low EF. Blood cultures remained negative, making sepsis unlikely, and Cr remained within normal limits, decreasing suspicion of poor metabolite/drug excretion. Although no life-threatening events occurred, lactic acid levels should be carefully monitored when starting linezolid therapy to avoid the adverse effects associated with LILA. 

Currently, the FDA approves linezolid therapy for a maximum of 28 days [5]. Retrospective cohort studies, such as Mao et al., have suggested that a prolonged course of linezolid, ≥28 days, increases a patient’s risk of developing LILA and other adverse effects [1]. However, our patient developed lactic acidosis after only one day of treatment, which resolved once linezolid was discontinued. Lactic acidosis can have profound hemodynamic consequences for patients, including death [4]. A mortality rate of 25% has been associated with patients with lactic acidosis in non-shock states, whereas those with shock had a mortality rate of 50% [2]. A meta-analysis study of 35 articles with 47 patients (28 males, 18 females, and one non-binary) was done, which found a 25.5% mortality rate associated with LILA. This study also showed that the duration of therapy had an effect on mortality rates. Mortality was measured at 7 days, 14 days, and 28 days, being lowest at 14 days with a mortality rate of 20% [1]. Although the specific duration of therapy did not influence mortality, the overall mortality associated with LILA was very high and warrants close monitoring. 

Lastly, the proposed mechanism of LILA is closely related to mitochondrial-derived complexes, leading many to suspect a genetic risk factor for increased LILA susceptibility. For example, two alleles, HLA-B*38:02 and HLA-DRB1*08:03, are strongly associated with the adverse side effect of induced agranulocytosis when starting anti-thyroid medication [2]. If a gene existed in LILA, prior screening before the initiation of linezolid treatment could prevent its onset. Currently, limited studies have shown a genetic association.

## Conclusions

In conclusion, lactic acidosis associated with linezolid therapy can be fatal. Although some retrospective cohort studies have illustrated the duration of linezolid therapy as a possible risk factor, our case example casts uncertainty to that theory. When starting linezolid therapy, systematic monitoring of lactate levels should be considered to detect the early onset of LILA and prevent further complications. This case study aims to inform clinicians of the potential harmful side effects associated with linezolid, as well as the understudied risk factors involved in LILA that are needed to prevent its occurrence. Future studies are necessary to evaluate these risk factors.
